# Flexible Metamaterial Quarter-Wave Plate and Its Application in Blocking the Backward Reflection of Terahertz Waves

**DOI:** 10.3390/nano13071279

**Published:** 2023-04-05

**Authors:** Jinhai Sun, Yong-Qiang Liu, Jining Li, Xutao Zhang, He Cai, Xianli Zhu, Hongcheng Yin

**Affiliations:** 1 National Key Laboratory of Scattering and Radiation, Beijing 100854, China; 2 Institute of Laser and Optoelectronics, School of Precision Instruments and Opto-Electronics Engineering, Tianjin University, Tianjin 300072, China

**Keywords:** terahertz wave, quarter-wave plate (QWP), metamaterial, flexible, isolation

## Abstract

A terahertz flexible metamaterial quarter-wave plate (QWP) is designed and fabricated using polyimide as the substrate in this paper, with a 3 dB axial ratio bandwidth of 0.51 THz and high polarization conversion efficiency and transmittance. The effect of the incidence angle on the polarization conversion performance of the QWP is discussed by measuring the transmissions at multiple incidence angles. The blocking effect of this QWP combined with a polarizer on the backward reflection of terahertz waves is investigated by terahertz time-domain spectral transmission experiments. By adjusting the angle of the QWP and polarizer with respect to the incident light in the optical path, a blocking efficiency of 20 dB can be achieved at a 20° incidence angle, with a bandwidth of 0.25 THz, a maximum blocking efficiency of 58 dB at 1.73 THz, and an insertion loss of only 1.4 dB. Flexible terahertz metamaterial QWPs and polarizers can effectively block harmful reflected waves in terahertz communication and other systems. They have the advantages of a simple structure, ultra-thinness and flexibility, easy integration, no external magnetic field, and no low-temperature and other environmental requirements, thus having broad application prospects for terahertz on-chip integrated systems.

## 1. Introduction

In recent years, with the increasing maturity of terahertz technology, terahertz waves have been widely used in many application systems, such as terahertz detection [1,2], sensing [3,4], communication [5,6], and radar [7,8]. Especially in the application of 5G communication [9,10] and the technological development of 6G communication [11,12] in the future, terahertz communication technology has been widely considered by countries all over the world. However, due to the lack of suitable unidirectional transmission devices, the terahertz echoes caused by the reflections and scattering in these systems bring a lot of noise to the system’s operation and even danger to the key components, such as the light sources and detectors, thus severely limiting improvements to the performance of terahertz systems [13]. Therefore, a device to block the backward reflection of terahertz waves is urgently needed to overcome this limitation and enhance the performance of terahertz application systems.

It is well known that the transmission isolation of electromagnetic waves in the microwave field can usually be achieved using devices such as isolators [14,15] and circulators [16]. In the field of optics, many attempts have been made to achieve optical isolation by means of metamaterials [17] and photonic crystals [18]. In addition, in the terahertz field, terahertz isolators based on magneto-optical materials [19,20], semiconductor materials [21,22], graphene [23,24], photonic crystals [25,26], and metasurfaces [27,28] have also been widely reported in recent years. However, all of these isolators have disadvantages, such as a high insertion loss, magnetic field requirement, narrow working bandwidth, and difficult manufacturing. Most of them have not been experimentally validated or implemented. Fortunately, the combination of a quarter-wave plate (QWP) and polarizer can make up for the above shortcomings to some extent. Mendis and colleagues first offered, in 2017, a special device consisting of a QWP and a polarization beam-splitter, both made of uniformly spaced metal plates, which can isolate backward terahertz waves with a 0.97 dB insertion loss and an over 52 dB isolation at 0.42 THz [29]. However, the device’s working bandwidth of over 20 dB is only 0.02 THz, and the metal plate QWP is large in volume, making it unfavorable for use in integrated systems. The weak response of conventional QWPs based on birefringent materials to terahertz waves leads to a high insertion loss, large volume, and narrow working bandwidth. In contrast, terahertz QWPs based on metamaterials have the advantages of a low insertion loss, small volume, and large bandwidth of a 3 dB axial ratio. Artificially designed metamaterials can have properties that ordinary materials in nature do not possess, so metamaterials are widely used in the terahertz band [30,31] to make up for the ineffective response of natural materials to terahertz waves. In addition to terahertz QWPs, the application of metamaterials in active terahertz devices [32,33] has gained much attention in recent years. Tunable terahertz metamaterial devices [34,35,36] proposed by several research groups have further expanded the application of metamaterials in the terahertz field. Currently, silicon and silicon dioxide are mainly chosen as the substrate materials for terahertz metamaterials [37,38], but the hardness of these substrate materials limits the application of waveplate integration. In 2011, Hong et al. began research on flexible metamaterials [39]. Flexible metamaterials are quickly applied to functional devices, such as terahertz sensors [40] and QWPs [41]. A flexible metamaterial QWP is characterized by high polarization conversion efficiency, small volume, easy integration, and low insertion loss. Therefore, backward terahertz wave blocking devices based on flexible metamaterials will integrate more easily into terahertz systems.

A terahertz flexible QWP with high transmittance and a broad working bandwidth was fabricated by photolithographing two U-shaped unit cell gold films between three layers of polyimide, combining the diversity of metamaterials and the flexibility of polyimide in this paper. It was also combined with a polarizer to achieve a blocking function for terahertz backward reflections. The QWP achieved an axial ratio of less than 3 dB in the 1.46~1.97 THz range, with a polarization conversion efficiency of more than 70% and a transmission coefficient of more than 0.85 in the 0.51 THz bandwidth. By placing the QWP and polarizer at 20° relative to the incident optical path, the effective blocking of backward reflected terahertz waves was realized experimentally in the range of 1.57~1.82 THz, with a blocking efficiency of 20 dB, a low insertion loss of approximately 1.5 dB, and an effective operating bandwidth of 0.25 THz. An ultra-high blocking efficiency of 58 dB was achieved at 1.73 THz, with only a 1.4 dB insertion loss. This study proposes an implementation scheme for blocking terahertz backward reflection, which has the advantages of low insertion loss, ultra-thinness, easy integration, and high blocking efficiency, and it will have good prospects for system applications.

## 2. QWP’s Design and Simulations

**Figure 1:**
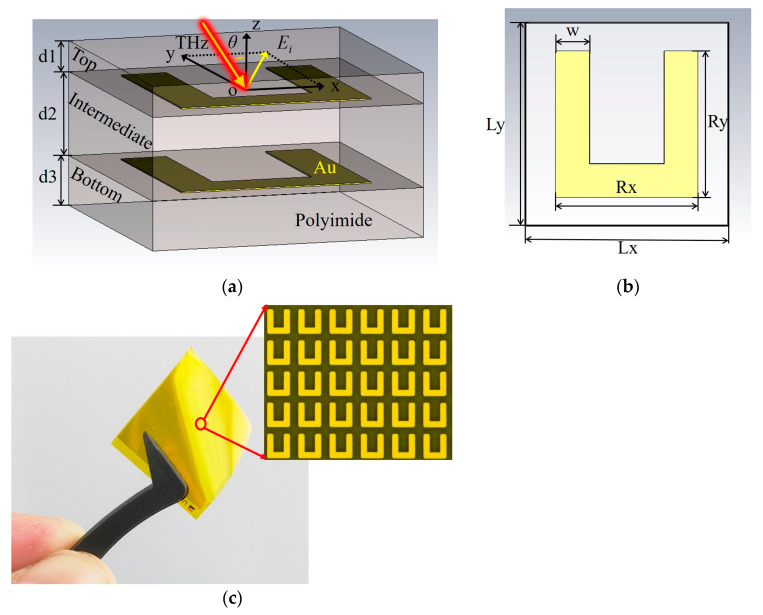
The structure of the designed QWP unit cell: (**a**) QWP unit cell structure designed using CST Microwave Studio; (**b**) structural dimensions of the U-shaped unit cell in the QWP; (**c**) a picture of the QWP in tweezers, where the upper right corner contains an image of the red circle area on the QWP, which has been magnified 1000 times.

Images of the designed unit cell structure and a sample of the QWP are provided in Figure 1. Figure 1a shows the three-dimensional structure of the QWP unit cell, which is composed of three dielectric layers and two metal films with the same pattern. The metal is gold, and its conductivity was set to 4.56 × 10^7^ S/m in the simulation. The thickness of the metal film is 0.2 μm. The dielectric layer is made of polyimide (PI). In the simulation, the relative permittivity and loss tangent of the PI were set to 2.8 and 0.0027, respectively [42]. The thicknesses of the three PI layers are d1 = 5 μm, d2 = 13.5 μm, and d3 = 8 μm, from the top to the bottom. The function of the two outer PI layers is to protect the metal structure, reduce reflection losses, and improve the transmission performance. Figure 1b shows the structural dimensions of the U-shaped unit cell in the QWP: Lx = Ly = 45 μm, Rx = 31.5 μm, Ry = 32.5 μm, and w = 7.5 μm. Figure 1c shows a picture of the QWP in tweezers, and the upper right corner contains an image of the red circle area on the QWP, which has been magnified 1000 times.

**Figure 2:**
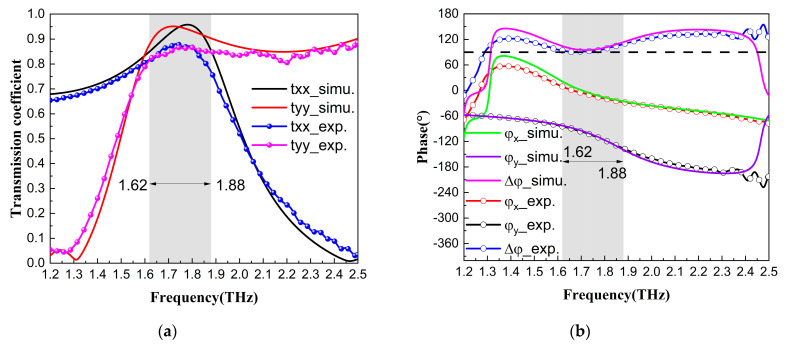
(**a**) Transmission coefficient and (**b**) phase and phase difference of the incident wave in both the x- and y-directions, where the solid lines are the simulation curves and the dotted, solid lines are the experimental curves.

The terahertz wave transmission characteristics of the metamaterial unit cell structure of the QWP shown in Figure 1a were simulated using CST Microwave Studio software. The setting of the unit cell boundary condition was applied in the x- and y-directions, and the open (add space) boundary condition was used in the z-direction. For comparison with the experiment, the phase shift of the thin film metamaterial device was the result relative to the air layer, the phase shift of which was set to 0° [43]. The air layer’s thickness was 26.5 μm, which was the same as the QWP’s thickness. As shown in Figure 1a, the incidence angle *θ* is the angle between the incident direction of the terahertz wave and the -z-axis in the xoz-plane. The transmission coefficients (*t_x_*, *t_y_*), phases (*φ_x_*, *φ_y_*), and phase difference (Δ*φ* = *φ_x_* − *φ_y_*) of the incident wave in both the x- and y-directions were obtained by simulation when the incidence angle was 0°, as shown by the solid lines in Figure 2. The dotted, solid lines in Figure 2 are the corresponding data obtained from the terahertz time-domain spectroscopy experiments. It can be seen that the simulation and experimental results are in good agreement. In the range of 1.62~1.88 THz, the transmission coefficients in both the x- and y-directions were kept above 0.85, and the phase differences were kept at approximately 90°. The inductor–capacitor (LC) oscillations near 1.3 THz were strong for the y-direction transmission terahertz waves, which leads to severe phase abruptness and energy loss of the terahertz waves. The interaction of the QWP structure with the terahertz waves was weaker near 1.7 THz, resulting in the high y-component transmission of the terahertz waves. The impedance matching of the metamaterial and air achieved by magnetic dipole resonance greatly improves the x-component transmission of the terahertz waves. The high transmittance in the x- and y-directions means that the structure has a very low insertion loss.

**Figure 3:**
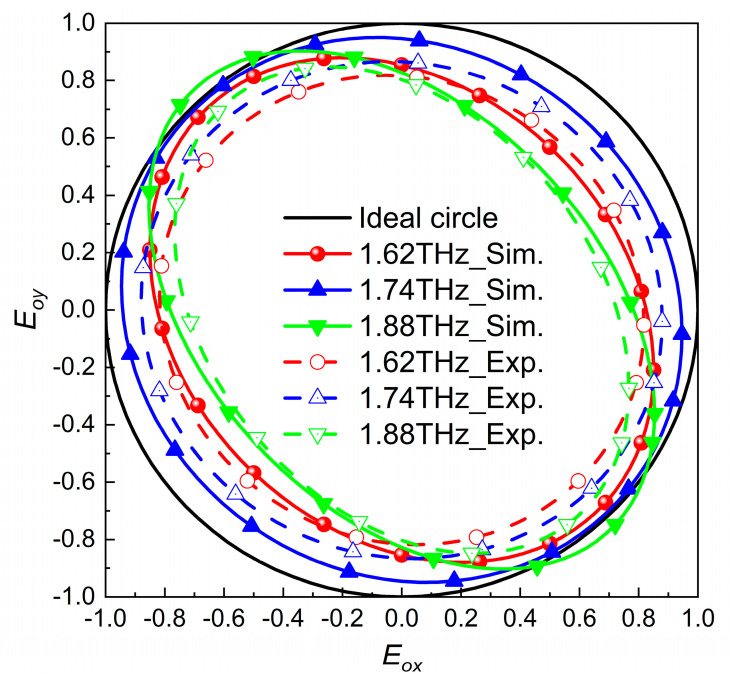
Circularly polarized wave simulation (dotted, solid lines) and experimental (dot–dashed lines) results.

The electric field of incident of the linearly polarized (LP) wave was incident on the QWP at an angle of 45° from the x- and y-directions and divided into the x- and y-components with same amplitude and phase. The two components were finally synthesized into the circular polarized wave by the QWP. Taking 1.62 THz, 1.74 THz, and 1.88 THz as examples, the long and short axes of the transmitted waves could be obtained by calculation, and the polarization ellipses obtained by simulation are shown as the dotted, solid lines in Figure 3. The dot–dashed lines are the measured polarization ellipses at these three corresponding frequency points, and it can be seen that all three frequency points present good circularly polarized waves.

## 3. Measurement Results of QWP Transmission Performance

With a typical terahertz time-domain spectroscopy system, the transmission of a 45° LP terahertz wave when it is vertically incident on this QWP can be measured, as shown by the dotted, solid lines in Figure 2a. A comparison of the experimental and simulation results shows that the working bandwidth was consistent, but the measured transmittance was slightly lower than the simulated results due to the presence of factors such as the influence of the actual PI material on the terahertz transmittance performance in the measurements.

**Figure 4:**
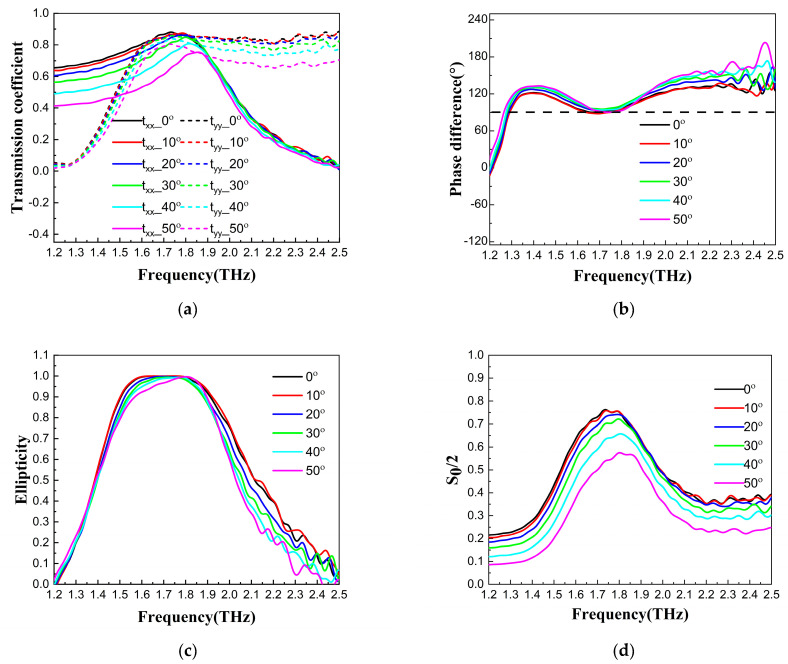
(**a**) Transmission coefficients, (**b**) phase differences, (**c**) ellipticities, and (**d**) *S*_0_/2 of terahertz waves in both the x- and y-directions at different incidence angles.

To investigate the effect of the incidence angle on the polarization conversion performance of the QWP, the transmission coefficients in both the x- and y-directions, ellipticities, phase differences, and transmittances of the QWP were measured at different incidence angles with a terahertz time-domain spectroscopy system, as shown in Figure 4. The transmission coefficients and ellipticities of the terahertz waves decreased obviously when the incidence angles were greater than 30°. The QWP polarization conversion performance can be described by the Stokes parameters with the following equations [44].

(1) $$S_{0}=t_{xx}^{2}+t_{yy}^{2}$$

(2) $$S_{1}=t_{xx}^{2}-t_{yy}^{2}$$

(3) $$S_{2}=2t_{xx}t_{yy}\cos\Delta \varphi$$

(4) $$S_{3}=2t_{xx}t_{yy}\sin\Delta \varphi$$

(5) $$\chi =\frac{S_{3}}{S_{0}}$$

(6) $$\beta =\frac{\arcsin\chi }{2}$$

The outgoing waves with an ellipticity of −1 and 1 are perfect left circularly polarized (LCP) and right circularly polarized (RCP) waves, respectively. *S*_0_/2 is the transmittance. When the incidence angle increased to 30°, the transmission coefficients in both the x- and y-directions decreased prominently, as shown in Figure 4a. When the incidence angle increased from 0° to 50°, the phase differences were close to 90° at approximately 1.7 THz, as shown in Figure 4b. The ellipticity curves of the LP wave along the diagonal direction of the QWP at an incidence angle from 0° to 50° are shown in Figure 4c. The experimental results of the transmittance at different incidence angles are shown in Figure 4d. *β* is the elliptic angle. The axial ratio *R* and the polarization conversion efficiency *η* can be calculated from the above Stokes parameters as follows.

(7) $$R=10\mathrm{lg}\left(\tan\beta \right)$$

(8) $$\eta =\frac{{\left|t_{xx}\right|}^{2}+{\left|t_{yy}\right|}^{2}}{2}$$

**Figure 5:**
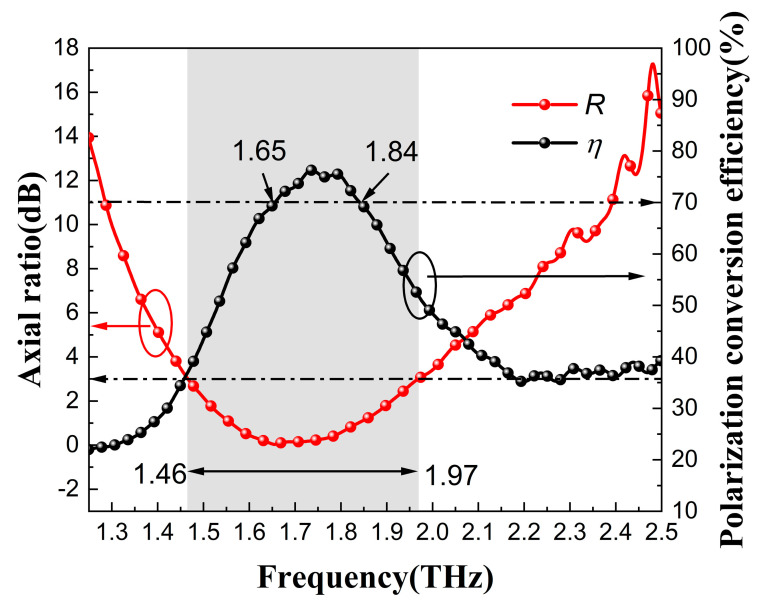
The axial ratio and polarization conversion efficiency of the QWP at an incidence angle of 0°.

The axis ratio can be used to describe the shape of the elliptically polarized wave. When the axial ratio is less than 3 dB, the transmitted wave can be regarded as a usable circularly polarized wave [45]. Figure 5 shows the axial ratio and the polarization conversion efficiency at a 0° incidence angle. It can be seen that the axial ratio was less than 3 dB in the range of 1.46–1.97 THz, the bandwidth was up to 0.51 THz, and the polarization conversion efficiency was more than 70% in the range of 1.65–1.84 THz.

## 4. Application of QWP in Blocking Backward Reflection

**Figure 6:**
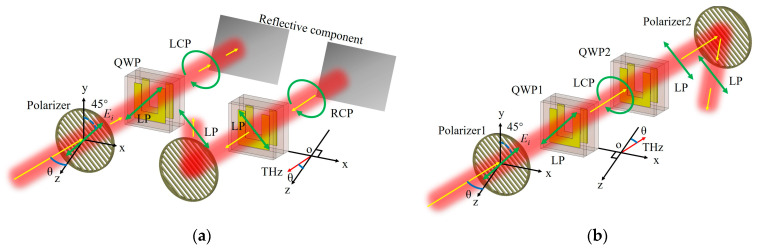
(**a**) Schematic diagram of the decomposition of the terahertz transmission optical path. On the left is the optical path of the terahertz wave entering the blocking device and reaching the reflective component, and on the right is the optical path of the terahertz wave being reflected by the reflective component and blocked by the polarizer. (**b**) Schematic diagram of the equivalent experimental optical path for measuring the blocking efficiency and insertion loss in this study.

The wire grid structure polarizer was made of multiple tungsten wires, with a pitch of 30 μm and a width of 10 μm. The transmittance of the polarizer was close to 1 and 0.005 in the polarization direction and perpendicular to the polarization direction, respectively. As shown in Figure 6, the polarizer is parallel to the QWP, and its wires are oriented along the diagonal direction of the QWP. *θ* is the incidence angle, i.e., the angle between the direction of the incident optical path and the -z direction perpendicular to the surface of the polarizer and the QWP. As shown in Figure 6a, the LP terahertz wave after passing through the polarizer, which has a polarization direction that is parallel to the diagonal of the QWP, is first converted into an LCP terahertz wave after passing through the QWP. Reflected by the reflecting component, the rotation direction of the terahertz wave electric field remains unchanged. However, due to the change in the propagation direction, it becomes an RCP terahertz wave with respect to the QWP to be passed. When the RCP terahertz wave passes through the QWP again it becomes an LP wave perpendicular to the polarization direction of the original incident LP wave. Therefore, after passing the QWP twice, the terahertz wave cannot pass through the original polarizer again. When the *θ* is not 0°, the polarizer reflects the terahertz wave out of the original optical path. It should be noted that the direction of rotation of the LCP wave remains the same when it is reflected, but it becomes an RCP wave with respect to the QWP as the propagation direction becomes opposite. The Jones matrix equation for determining the polarization state is as follows.

(9) $$E_{r}=TG_{1}G_{2}G_{1}E_{in}=\left[\begin{array}{cc} -1 & 0\\ 0 & 1 \end{array}\right]\left[\begin{array}{cc} 1 & 0\\ 0 & -i \end{array}\right]\left[\begin{array}{cc} -1 & 0\\ 0 & 1 \end{array}\right]\left[\begin{array}{cc} 1 & 0\\ 0 & -i \end{array}\right]\left[\begin{array}{c} 1\\ 1 \end{array}\right]=\left[\begin{array}{c} 1\\ -1 \end{array}\right]$$

where *E_in_* denotes the transmission matrix of the polarizer; *G*_1_ denotes the transmission matrix of the QWP, whose fast axis is along the y-direction; and *G*_2_ denotes the transmission matrix of the reflective component. Since the reflected beam propagates in the opposite direction to the incident beam, this results in a transformation matrix *T* in the coordinate system. According to the matrices of *E_in_* and *E_r_*, the electric field direction of the final reflected LP terahertz wave is perpendicular to that of the incident LP terahertz wave, so the reflected terahertz wave cannot pass through the original polarizer. In view of the optical path limitation in the terahertz time-domain spectroscopy measurement and the convenience of the transmission experiment relative to the reflection experiment, as shown in Figure 6b, the transmission of two QWPs placed in the same tilting direction is measured in the actual experiment. The blocking effect is the same as shown in Figure 6a, and the Jones matrix equation for Figure 6b is as follows.

(10) $$E_{t}=G_{1}G_{1}E_{in}=\left[\begin{array}{cc} 1 & 0\\ 0 & -i \end{array}\right]\left[\begin{array}{cc} 1 & 0\\ 0 & -i \end{array}\right]\left[\begin{array}{c} 1\\ 1 \end{array}\right]=\left[\begin{array}{c} 1\\ -1 \end{array}\right]$$

**Figure 7:**
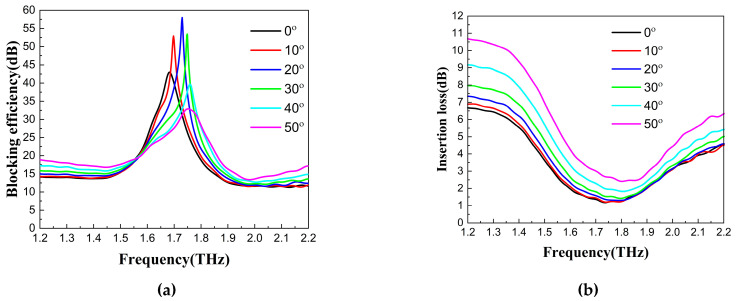
(**a**) Measured blocking efficiency curves and (**b**) insertion loss curves of this blocking device at different incidence angles.

From Equations (9) and (10), it can be seen that the final reflected electric field and transmitted electric field are the same, so the two methods are equivalent. According to the equivalent method in Figure 6b, the transmittances of LP terahertz waves at different incidence angles are measured by passing two QWPs consecutively. The blocking efficiency (i.e., the ratio of the outgoing light power from the blocking device to the light power entering the blocking device) and the insertion loss of this blocking device are calculated in Figure 7a,b, respectively. As can be seen in Figure 7a, the blocking efficiency varied with the incidence angle when the polarizer and QWP were always kept parallel to each other in use. There were working frequency points with optimal blocking efficiency. From Figure 7b, we can see that the frequency point where the minimum insertion loss was located gradually blue-shifted with the increase in the incidence angle. The insertion loss increased with an increasing incidence angle. Mastering the variation law of the QWP’s blocking efficiency and insertion loss helps to achieve its best performance in practical applications.

It can be found from Figure 7 that the optimal blocking frequency points, blocking efficiency, and insertion loss are different for different incidence angles. From the experimental results, the blocking effect is more ideal at a 20° incidence angle, reaching a maximum blocking efficiency of 58 dB at 1.73 THz, while the insertion loss is only 1.4 dB. The blocking efficiency exceeds 20 dB in the 1.57–1.82 THz range, with a blocking bandwidth of 0.25 THz, while the insertion loss is approximately 1.5 dB. When the incidence angle is 10°, the blocking efficiency exceeds 20 dB in the range of 1.57–1.80 THz, with a blocking bandwidth of 0.23 THz, and the maximum blocking efficiency is 57 dB at 1.7 THz, while the insertion loss is approximately 1.4 dB. The experimental results show that the insertion loss is smallest at an incidence angle of 10°, and the blocking effect is best at an incidence angle of 20°.

## 5. Conclusions

A flexible terahertz QWP based on metamaterial was designed and fabricated with a 3 dB axial ratio bandwidth of 0.51 THz in the range of 1.46–1.97 THz. The polarization conversion efficiency was greater than 70% in the range of 1.65–1.84 THz, as measured experimentally. The backward terahertz reflection blocking device composed of the terahertz QWP and the metal wire grid polarizer could block the backward terahertz reflection with a blocking efficiency of more than 20 dB within a 0.25 THz bandwidth when placed at an incidence angle of 20°, while the insertion loss was only approximately 1.5 dB. The maximum blocking efficiency occurred at 1.73 THz, with a blocking efficiency of 58 dB and an insertion loss of 1.4 dB. Thus, this combination achieves the effective blocking of backward terahertz reflections while ensuring a forward transmission of sufficiently high energy.

We compared the blocking efficiency and insertion loss of several devices and solutions with backward reflection blocking or isolation capabilities in the terahertz frequency band, as shown in the table below.

As can be seen from Table 1, the combination scheme of the U-shaped QWP and wire grid polarizer makes full use of the advantages of the metamaterials and flexible materials, such as ultra-thinness, easy integration, high blocking efficiency, and low insertion loss. Therefore, it has broad application prospects in future terahertz communication, radar, and other application systems.

## Figures and Tables

**Table 1:** Comparison of the blocking efficiency and insertion loss of several terahertz devices and solutions with backward reflection blocking or isolation capabilities.

Time	Frequency	Author	Structure	Blocking Efficiency	Insertion Loss
2015	Tunable	Chen [46]	THz isolator with InSb	43 dB	1.79 dB
2016	2.9 THz	Tamagnone [47]	THz isolator with graphene	20 dB	7.5 dB
2017	0.42 THz	Mendis [29]	QWP and polarizer made with metal plates	52 dB	0.97 dB
2018	Tunable	Lin [48]	THz isolator with InSb	35 dB	6.2 dB
2020	106.6 GHz	Portela [49]	Photonic crystal	15 dB	1.68 dB
2020	1.2 THz	Keshock [50]	Faraday isolator with InSb	18.8 dB	12.6 dB
2020	2.136 THz	Ji [51]	THz isolator with InSb	55 dB	3.92 dB
2021	0.47 THz	Yuan [52]	On-chip isolator with InSb	52 dB	7.5 dB
2022	1.51 THz	Sun [53]	H-shaped metasurface QWP and polarizer	50.7 dB	1.65 dB
2023	1.73 THz	This paper	U-shaped metamaterial QWP and polarizer	58 dB	1.4 dB

## Data Availability

The data presented in this study are available on request from the corresponding author.
